# The role of gender in peer-group perceptions of climate scientists’ media statements

**DOI:** 10.1177/09636625211029198

**Published:** 2021-07-02

**Authors:** Lauren Armstrong, George Adamson

**Affiliations:** King’s College London, UK

**Keywords:** gender and science, media and science, science attitudes and perceptions, science communication, scientists - attitudes

## Abstract

This research explores whether environmental scientists perceive their male and female peers differently when making statements in the media including policy advocacy. Environmental scientists in the United Kingdom were provided with a media statement by a fictitious scientist containing a mixture of scientific information and advocacy, and asked to rate the statement against various attributes. Attributes were designed to represent stereotypes associated with male and female tendencies, and with science (impartial objectivity) and the media (dramatic narrative). The statements were randomly assigned to one of two male and two female scientists. Where the statements were attributed to a female scientist, male environmental scientists rated the fictitious scientist as significantly more ‘dramatic’ and ‘biased’ than their female counterparts did. These gendered attributes are typically held as contrary to the norms of science, suggesting an implicit bias among male scientists when reviewing their female peers’ media statements.

## 1. Introduction

The close coupling of climate science with policy has convinced a number of climate scientists to advocate for particular policy options when conducting interviews in print, online or screen media (Brownell et al., 2013). While there are several reasons why a scientist may choose whether or not to make such statements – including differences in opinion on the role that scientists should play in society, and inconclusive evidence on the efficacy of scientists’ advocacy in engendering climate change mitigation (Boykoff and Oonk, 2020; Nelson and Vucetich, 2009; Schmidt, 2015) – many scientists that are convinced of the importance of advocacy refrain from doing so. Lack of engagement can be either due to a lack of communication training (Brownell et al., 2013), or a perception that such activities will have implications on their credibility (Miyakawa, 1999; Polcin, 2014). There can be a fear that media statements including advocacy will be construed as at odds with the objectivity of science, and hence reduce scientists’ credibility within their peer group and wider society (Barbalet, 2002; Brysse et al., 2013). These fears are a particular concern for female scientists; it has long been recognised that women face greater challenges than their male counterparts within the scientific community (Moss-Racusin et al., 2016). Many science norms are considered as ‘masculine’ traits, and socio-cultural ‘female’ stereotypes are typically associated with the more emotive and theatrical requirements of communication and the mainstream media, not science (Brysse et al., 2013). Faced with pre-existing biases, therefore, female scientists may avoid advocacy or media appearances altogether, due to a perception that such activity will heighten a perception among their peers that their activities run contrary to the norms of science. However, while the implications of advocacy on the credibility of scientists within society has received previous attention (Blockstein, 2002; Donner, 2014; Horton et al., 2016; Kotcher et al., 2017), peer group perceptions have not.

This study explores the role of gender in informing peer-group (i.e., other environment scientists’) perceptions on scientists advocating for particular policies in their media appearances. The study seeks to understand whether environmental scientists as a community rate other scientists’ media statements differently based on their gender. It also explores whether scientists’ own gender affects their perceptions of other male or female scientists making statements in the media. Note that there can be considerable difficulty in defining advocacy and disentangling it from communication of science (Spence and Pidgeon, 2010). Justifying a project’s relevance to society to secure funding, or use of terms such as ‘degradation’ or ‘conservation’ can implicate a preferred policy outcome (Lackey, 2007; Schmidt, 2015). In this paper, we consider advocacy to be ‘any communication that supports a policy, cause or action’.

### Gender, media, and advocacy

Unlike more salient environmental concerns such as plastic pollution – which is directly visible and grants immediate efficacy when action is taken (Syberg et al., 2018) – climate change is ‘invisible’ and distant. Humans cannot directly see climate change occurring, and it is monitored over large temporal and spatial scales (Sheppard, 2012). Climate science is also complex and uncertain. Climate scientists are regularly called upon to make climate change ‘visible’ through popular media, including giving detailed explanations of the findings of climate science and discussing the links between particular weather events and anthropogenic climate change (Boykoff and Boykoff, 2007). Within these media appearances, scientists are often asked to comment on particular policy options or economic models to mitigate against climate change. While the expertise required to understand, for example, energy transitions are very different to the majority of climate scientists’ training, many scientists choose to directly respond to these questions. Studies have shown that scientists are generally trusted in society (Corner et al., 2018), and some studies have suggested that consistent scientific messaging can increase support for climate policy (Van Der Linden et al., 2015). Many climate scientists consider involvement in policy dialogues a moral obligation, due to the risks posed by climate change (Nelson and Vucetich, 2009), and consider advocacy an expression of their rights as concerned citizens who also happen to be scientists (Kaiser, 2000).

The question of if and how scientists should play a role in policy dialogues, however, remains a pervasive point of discussion in the scientific community (Boykoff and Oonk, 2020; Kotcher et al., 2017). Science has been professionalised through values and norms which have given it authority and status (Jasanoff, 1987). These norms, such as logic, objectivity, level-headedness and scepticism, are seen to support credibility by rigorously ensuring robust scientific method, key to obtaining valid and accurate knowledge (Barbalet, 2002; Merton, 1973). Adhering to such norms allows scientists to avoid losing standing within peer groups and the public (Brysse et al., 2013). It is often supposed that advocacy in media will harm credibility, based on the premise that a scientist should be an objective observer, whereas advocacy is linked to subjective judgements on how the world should be (Kotcher et al., 2017; Nelson and Vucetich, 2009). Media also value narrative or story-based communication (Hillier et al., 2016), emotive messages, simplified complex language and a focus on dramatic aspects (Moser, 2010), which run counter to the objective, non-personalised language of science.

The risk of being regarded as dramatic or emotional is particularly great for female scientists (Brysse et al., 2013). Men are more often stereotyped with *agentic* traits, which are related to self-assertion and independence. These traits are more readily associated with the norms of science (Knobloch-Westerwick et al., 2013), and Ramsey (2017) shows that academics tend to associate these agentic qualities with academic success. Stereotypical female characteristics tend to be associated with *communal* traits, characterised as sensitive or emotive (for example ‘caring’ and ‘dramatic’) (Fox, 2006). These disparities have been associated with unintentional or subtle bias, as a result of unconscious or implicit processes (Moss-Racusin et al., 2012). These unconscious processes are factors of gender stereotypes, schemas and pervasive socio-cultural norms (Easterly and Ricard, 2011; Heilman, 2012; Staats et al., 2015).

Pre-existing gender biases in science therefore present a particular barrier to female scientists in their media appearances, alongside other challenges faced by women in science (Bostock, 2014; Cislak et al., 2018; Handelsman et al., 2005; Moss-Racusin et al., 2012, 2016). Women receive less support when considering entry to PhD areas, lower evaluations for writing skills and journal articles (Gannon et al., 2001), fewer citations (Larivière et al., 2013), lower funding success (Bornmann et al., 2007), lower pay and slower career progression (Ceci and Williams, 2007). These differences in expectations account for the Matilda effect, in which there is a systematic under-recognition of female scientists within scientific institutions. Knobloch-Westerwick et al. (2013), for example, found that identical conference abstracts were more positively evaluated when attributed to a male author over a female author. The ultimate outcome of these biases is to reduce diversity throughout academia (Handley et al., 2015). In a comprehensive review of gender-bias research, Ceci et al. (2009) concluded that, although the reason for fewer women in science is predominantly a result of personal choice, these choices are subject to pervasive limiting factors and socio-cultural norms, with even small biases accumulating into the disparities seen in academia.

Studies have shown that gender-based career stereotypes can be countered by increasing the visibility of female scientists, facilitating normalisation of women in science (Kitzinger et al., 2008). Social cognitive theory has shown that learning occurs via social reinforcement and that people observe and copy those they identify with. Similarly, recursive causality suggests that strategic objectives interact via feedback mechanisms, where an initial success generates conditions for additional success (Rogers, 2008). As such, Stout et al. (2011) found that even short exposure to female scientists increased women’s perceived fit to, and interest in science. Mainstream media has particularly shown a demonstratable effect in influencing ‘normality’ and aiding social change (Buck et al., 2008). Therefore, perceived barriers to media appearances due to fears of gender-based judgements by their peers can actually serve to strengthen gender stereotypes in science.

Greater involvement of relatable female messengers can help enhance female engagement with particular issues (Jones, 2014; Pearson et al., 2017). Diversity in environmental communication can enhance public engagement via relatable messengers, such as those who share the same gender (Pearson et al., 2017). Diversity of messengers is a particularly important within climate change, where women tend to underestimate their understanding relative to men (Ballew et al., 2018). Greater diversity of voices can limit domination of the issue by certain groups, which influences questions posed and interpretation of results, leading to homogeneity of ideas and interpretation (Clark-Blickenstaff, 2005; Page, 2007). Women are also more likely to be adversely impacted by climate change than men in many countries, and as such the UN Sustainable Development Goals recognise gender equality as an essential method to combat climate change. Having role models that engage women worldwide while recognising women as decision-makers, experts and stakeholders, would therefore likely enhance the building of successful solutions, with opportunity for large scale impact (International Union for Conservation of Nature (IUCN), 2015). Therefore, examining potential barriers to greater visibility of women in climate science and policy has significant implications.

## 2. Methodology

Data collection for this study used online-surveys, incorporating predominantly closed-ended questions with an option for open-ended expansion via a comments box. Surveys were developed on the open-source LimeSurvey software and sent to environmental scientists with at least PhD-level education, considered to be representative of climate scientists’ peers. The target population was chosen to represent the peer-group of climate scientists, given the multi-disciplinary nature of climate change; that is, climate research comes from many areas of environmental sciences. Relevant universities were found on-line. Email addresses (n = 1465) were obtained through university websites, with online research profiles read to ensure relevance to environmental science. The full list of universities is provided in the Supplemental Material, section 1.0. Participants received an email containing the topic overview, an invitation to take part, a link to an online survey and an information sheet. To reduce the possibility for social desirability bias, participants were not informed in advance that the survey would be looking at gender differences, instead being told that the study was ‘investigating peer-group perceptions of climate change advocacy among environmental scientists in the UK’. High-risk ethical approval was obtained from the King’s College London Research Ethics Committee.

**Figure 1.**
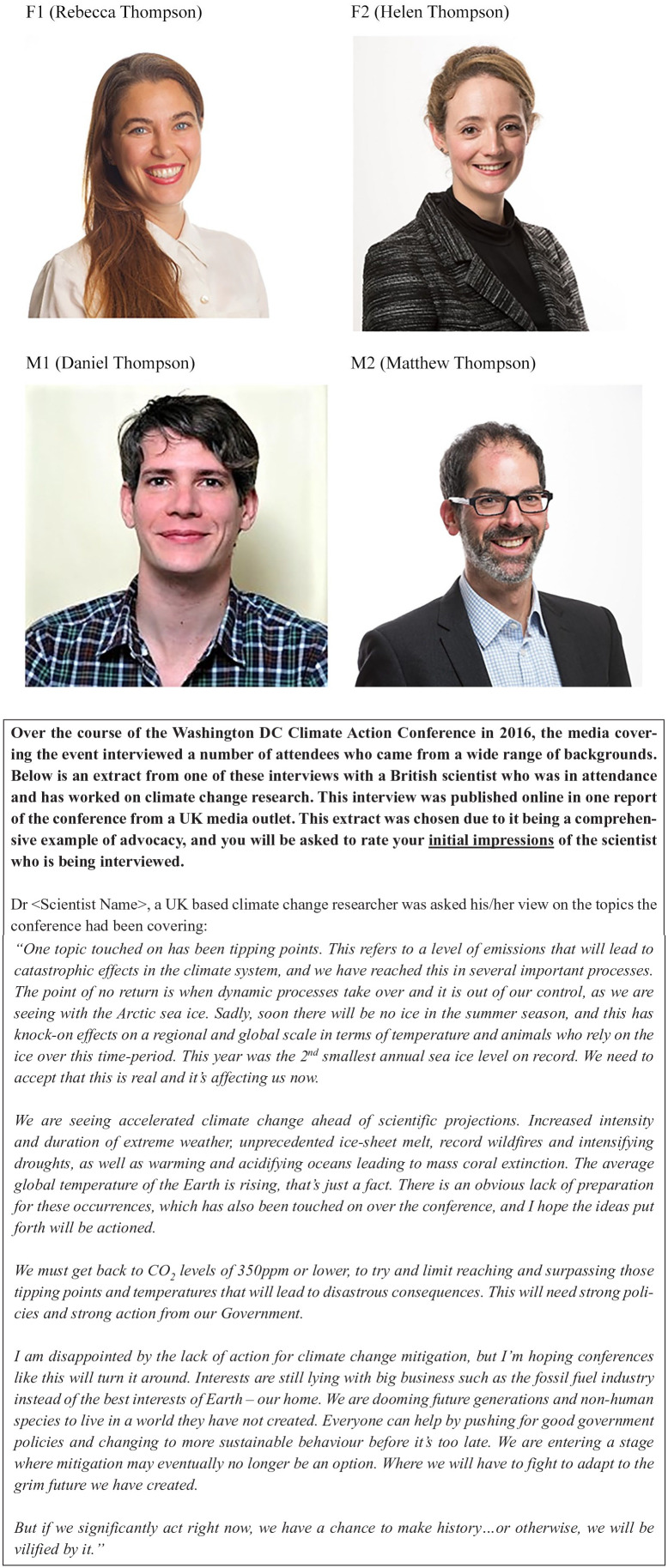
Four fictitious scientist’s images and names, together with accompanying text.

The survey includes three sections (see Supplemental Material section 2.0 for full survey): Demographic Information (section 1), the Media Statement (section 2) and information on the Roles of a Scientist (section 3, not analysed here due to small sample size within each category). Section 1 collected demographic information including gender, education level and participant ethnicity. In section 2, participants were presented with a fabricated interview with a British scientist associated with a real conference – the Climate Action 2016 Summit. The interview was attributed to one of four fictitious scientists selected at random, two male and two female (‘the scientists’). Figure 1 shows the text of the interview and the images of the four fictitious scientists. All participants were presented with the same text, designed to reflect a typical media statement by a climate scientist containing a mixture of scientific information and advocacy. Participants were unaware that they were only seeing one scientist and that the situation was fabricated. Names were chosen based on popular baby names from 1984 and the top surnames in the United Kingdom. Photos were obtained with permission from the researchers’ network, using people of similar ethnicity and age to try and eliminate variables outside of gender.

After reading the media statement, participants were asked to rate the fictitious scientists on 23 attributes. Each attribute was selected from the literature to represent an underlying stereotypical association. These associations were ‘male’ (for example competitive, decisive), ‘female’ (emotive, caring), those associated with ‘science’ (objective, impartial), and the narrative style found in ‘media’ (dramatic, biased). Attributes and the literature from which they were selected are presented in Table 1. Participants were also asked to rate how accurate they found the piece. Rating employed a 10-point Likert-type scale from ‘not at all’ to ‘extremely’, as used by Knobloch-Westerwick et al. (2013). For the purpose of analysis, the Likert-type scales for the individual attributes were also combined to give four Likert-type scales for ‘Male’, ‘Female’, ‘Science’ and ‘Media’ attributes, using median values for inferential analysis. All combined scales were tested for internal consistency and reliability using Cronbach’s alpha and factor analysis. The reliability coefficients returned were all > 0.7, pertaining to a good level of internal reliability for each scale (Tavakol and Dennick, 2011). Factor analysis highlighted ‘dispassionate’ and ‘competitive’ as not being unidimensional for the ‘Science’ scale, so these were removed from analysis.

**Table 1.** Attribute types and corresponding stereotypical association. The final column displays the literature this was taken from.

Attribute	Association				Reference
	‘Male’	‘Female’	‘Science’	‘Media’	
Objective	X		X		Heilman (2012); Brysse et al. (2013)
Restrained	X		X		Heilman (2012); Brysse et al. (2013)
Emotive		X		X	Heilman (2012); Brysse et al. (2013)
Dramatic		X		X	Moser (2010); Brysse et al. (2013)
Competent	X		X		Knobloch-Westerwick et al. (2013)
Competitive	X		X		Fang and Casadevall (2015)
Decisive	X				Heilman (2012)
Sincere		X			Rudman and Goodwin (2004)
Rational	X		X		Barbalet (2002); Heilman (2012)
Dispassionate			X		Turbek et al. (2016)
Self-controlled	X		X		Brysse et al. (2013); Carli et al. (2016)
Level-headed	X		X		Brysse et al. (2013); Carli et al. (2016)
Impersonal			X		Fischer (2000); Fox (2006)
Credible			X		Brysse et al. (2013); Knobloch-Westerwick et al. (2013)
Caring		X			Carli et al. (2016)
Sceptical			X		Brysse et al. (2013)
Expressive		X		X	Heilman (2012); Moser (2010)
Warm		X			Rudman and Goodwin (2004)
Trustworthy			X		Corner et al. (2018)
Approachable		X			Heilman (2012)
Logical	X		X		Barbalet (2002); Brysse et al. (2013)
Impartial	X		X		Turbek et al. (2016)
Biased				X	Boykoff (2011)

Permutation tests were undertaken to compare groups. These tests were selected to minimise Type I testing errors, which inflate with multiple testing (Camargo et al., 2008; Chen et al., 2017). Other multiple test correction methods such as Bonferroni correction were deemed to be overly conservative (Nakagawa, 2004). To overcome the limitations of showing p-values alone (cf. Wasserstein and Lazar, 2016), common language effect sizes are also presented. Confidence intervals on common language effect sizes were calculated using a bootstrapping method.

## 3. Results

Out of 1465 potential participants, 91 completed surveys were received, a response rate of 6%. Most participants identified as male. Supplemental Material Section 3.0 provides a breakdown of the participant demographics. Female participants made up 25.3% of responses, with 12.1% giving no gender. Demographics of responses were similar to the target population of British academics as recorded by the Higher Education Statistics Agency for 2017/18 (Higher Education Statistics Agency (HESA), 2019). In all, 67.1% of respondents (n = 61) identified as White – British. 73.6% (n = 67) of respondents were at lecturer level or above, with 23.1% (21) postdoc and 3.3% (3) PhD candidates. Due to the low diversity of participant ethnicity and career stage, these factors were not accounted for the in the analysis. 21 participants were randomly assigned Male Scientist 1 (M1), 16 Male Scientist 2 (M2), 27 Female Scientist 1 (F1) and 27 Female Scientist 2 (F2). In total, 37 respondents were assigned male scientists (Male Combined – MC) and 54 female scientists (FC).

### Gender perception

To assess if attribute perceptions significantly changed based on gender, individual attributes were analysed between FC and MC, and individually between M1, M2, F1 and F2 (see Supplemental Material Table S2). There was no evidence that participants systematically rated the fictitious scientists differently on any attribute. Results were further analysed using the combined Likert-type scales, to represent the stereotypical associations (Table S3). M2 was rated as significantly higher on ‘Science’ attributes than F1 (p = 0.004; Y > X = 52% (46%–56%) Y < X = 35% (30%–41%) and M1 (p = 0.006; Y > X = 53% (47%–58%) Y < X = 33% (28%–39%) No other analyses produced p values ⩽ 0.1.

Results for MC and FC were also analysed to account for differences between the participants’ genders (Table 2). Female participants rated the female scientists (FC) significantly higher than male participants did for ‘self-controlled’ (p = 0.09; Y > X = 68% (51%–84%) Y < X = 21% (4%–36%) although this is significant only at p ⩽ 0.1. Conversely, male participants rated the female scientists significantly higher than female participants did for ‘biased’ (p = 0.05; Y > X = 29% (11%–45%) Y < X = 59% (39%–77%) and ‘dramatic’ (p = 0.03; Y > X = 31% (13%–47%) Y < X = 58% (37%–77%) In addition, female participants rated the male scientists (MC) significantly higher than the male participants did for ‘approachable’ (p = 0.07; Y > X = 64% (40%–87%) Y < X = 20% (1%–41%) and ‘expressive’ (p = 0.09; Y > X = 59% (35%–81%) Y < X = 28% (5%–50%), although again this was significant only at p ⩽ 0.1. No attempt was made to assess the effect of participant gender for individual scientists due to the small sample size.

**Table 2.** Median rankings of the twenty-three attributes assigned to the male (MC) and female (FC) climate scientists, by male (X) and female (Y) participants in the study. P values of permutation tests and common language effect sizes are provided where permutation tests produced p ⩽ 0.1.

Attributes	Median Rankings – MC		Median Rankings – FC	
	Male participant	Female participant	Male participant	Female participant
Objective	7	7	7	8
Restrained	5	4.5	5	6
Emotive	8	8	7	6
Dramatic	7	6	6.5	4^1^
Competent	8	8	8	8
Competitive	2	2.5	4.5	5
Decisive	7	8	7	8
Sincere	8	9	8	9
Rational	8	8	8	8
Dispassionate	3	2	3.5	3
Self-controlled	6	5.5	6	8^2^
Level-headed	7	6.5	6	8
Impersonal	3	2.5	4	5
Credible	8	8	8	9
Caring	8	9	8	8
Sceptical	2	2.5	3	3
Expressive	7	8.5^3^	7	7
Warm	7	7	5.5	6
Trustworthy	7	8	7	8
Approachable	7	9^4^	7.5	7
Logical	8	8	8	8
Impartial	5	4.5	5	5
Biased	4	4.5	4.5	2^5^

MC: male combined; FC: female combined.

1 p = 0.03; Y > X = 31% (13%–47%), Y < X = 58% (37%–77%), Y = X = 11%.

2 p = 0.09; Y > X = 68% (51%–84%), Y < X = 21% (4%–36%), Y = X = 11%.

3 p = 0.09; Y > X = 59% (35%–81%), Y < X = 28% (5%–50%), Y = X = 13%.

4 p = 0.07; Y > X = 64% (40%–87%), Y < X = 20% (1%–41%), Y = X = 16%.

5 p = 0.05; Y > X = 29% (11%–45%), Y < X = 59% (39%–77%), Y = X = 12%.

## 4. Discussion

The results of this study present no evidence to suggest that environmental scientists as a community systematically perceived their peers’ statements differently based on gender. These findings can contribute to the conversation about gender perceptions and equality in academia, and advocacy. ‘Visibility’ of scientists is increasingly being pushed as a normative precedent by universities, journals and the media (Franzen, 2012; Peters, 2012, 2013). While there are many reasons why a scientist might choose to make themselves more visible or not, this study suggests that fear of gendered judgement by their peer group as a whole should not put off female scientists from engaging in media activity, including policy advocacy. Encouraging female scientists to take on these visible roles, without fear of gender-based repercussions, could help foster conditions for change that could influence other factors of gender equality (Kalpazidou Schmidt and Cacace, 2017).

Nevertheless, these findings should be treated with caution, and are only valid when treating the participants as a population of men and women. When differentiating the participants by gender, our results do present some evidence that female environmental scientists rate both their female and male peers differently to their male counterparts. Female participants rated the two female scientists as somewhat more self-controlled than male participants did, in line with Carli et al.’s (2016) finding that female scientists associated women with more agentic qualities than male scientists did. In addition, Smyth and Nosek (2015) found that women in science tended to have weaker implicit science-male stereotypes, reflected here in the higher rating for the stereotypically ‘female’ attributes of approachability and expressiveness that female participants assigned to the male scientists, compared to their male counterparts.

More significantly, male participants rated the female scientists as significantly more biased and dramatic than the female participants did, both of which are stereotypically seen as being contrary to the norms of science. This finding is consistent with similar studies on gender-based stereotypes of those in leadership positions, with female leaders generally considered as more dramatic and more prone to making emotionally-driven judgements (Brescoll, 2016; Fischbach et al., 2015). It is also notable that these attributes are associated with media coverage of science, which scientists generally view as negative (Besley and Nisbet, 2013). This may therefore suggest a deeper underlying bias, with male scientists perceiving their female peers as having insufficient ‘scientific’ attributes to overcome the inherent issues with media coverage of science. Further study would be useful to see whether the finding is replicated across a population of non-scientists reading scientists’ media statements, as this could have implications for the way that media statements by female scientists are interpreted, and hence whether or not they choose to advocate for a particular policy position.

It should be noted that, although this study aimed to limit social desirability bias by veiling the study’s true aim until participants had completed it, one participant replied to the initial invitation saying that had guessed the true nature of the study but had tried to answer as honestly as possible. If more participants guessed the study’s aim and had responded in what they perceived to be a socially desirable manner, this could have contaminated the relationship between variables.

## 5. Conclusion and further work

This is the first study that has examined perceptions of climate scientists’ media statements by their own peers, where the statements contain a mix of scientific information and advocacy. In general, while the results suggest that scientists wishing to provide media statements including advocacy should not be put off through fear of judgement by their peers as a whole, there is some evidence that male participants associate media statements made by female scientists as significantly more biased and dramatic than female participants did. This finding merits further investigation, particularly to see if it is replicated in a wider population of readers beyond science.

Data for this study were collected within a specific socio-cultural location (the United Kingdom) and with a specific group of scientists (environmental scientists), so it would be unwise to apply these interpretations beyond these settings. Further work could include repeating the analysis with different scientist demographics such as ethnicity, which was not possible in this study due to the relatively small ethnic diversity of participants. Collecting information on participants’ disciplines would also be beneficial to investigate any disciplinary differences in peer-group perceptions of advocacy. In addition, ascertaining if variations in attribute perceptions translate into real-world behavioural differences would be valuable; for example, being more helpful in an advisory setting or championing to a greater degree professionally. Further studies could look at relationship strengths and networks between and within genders and advocacy views. Finally, this study utilised only four scientists, each of whom gave the same fictional statement. A greater range of scientists, and comparison between different forms of ‘advocacy’ through different wordings of the statements and differing ratios of ‘scientific’ versus ‘advocacy’ content, would be useful.

## Supplemental Material

sj-pdf-1-pus-10.1177_09636625211029198 – Supplemental material for The role of gender in peer-group perceptions of climate scientists’ media statements Click here for additional data file. Supplemental material, sj-pdf-1-pus-10.1177_09636625211029198 for The role of gender in peer-group perceptions of climate scientists’ media statements by Lauren Armstrong and George Adamson in Public Understanding of Science
