# Supplementary material for: The role of gender in peer-group perceptions of climate scientists’ media statements
Source: Public Underst Sci. 2021 Jul 2;30(7):841–53. doi: 10.1177/09636625211029198 (PMC8488648; doi:10.1177/09636625211029198)
Supplement: sj-pdf-1-pus-10.1177_09636625211029198 – Supplemental material for The role of gender in peer-group perceptions of climate scientists’ media statements [file sj-pdf-1-pus-10.1177_09636625211029198.pdf]

The role of gender in peer-group perceptions of climate scientists' media statements

Supplemental materials

Lauren Armstrong and George Adamson*

Department of Geography, King's College London

*Corresponding Author

Contents

1.0 List of Universities Consulted 2

2.0 Online Survey Questions 3

2.1 Survey Introduction 3

2.3 Media Statement 5

2.3.1 Male Scientist One (M1) – Daniel Thompson 5

2.3.2 Male Scientist Two (M2) – Matthew Thompson 6

2.3.3 Female Scientist One (F1) – Rebecca Thompson 7

2.3.4 Female Scientist Two (F2) – Helen Thompson 8

2.4 The Roles of a Scientist 9

2.5 Debrief 10

3.0 Breakdown of Results: Demographic Information 11

4.0 Breakdown of Results: Gender Perceptions 12

1.0 List of Universities Consulted

Aberystwyth University
Bangor University
Birkbeck University, London
Bournemouth University
Cardiff University
Durham University
Edge Hill University
Liverpool Hope University
Keele University
Kingston University London
London School of Economics (LSE)
Loughborough University
Newcastle University
Nottingham Trent University
Open University
Queen's University Belfast
Royal Holloway University
Swansea University
University of Aberdeen
University of Bath
University of Birmingham
University of Brighton
University of Bristol
University of Cambridge
University of Chester
University of Cumbria
University of Dundee
University of Edinburgh
University of Essex
University of Exeter
University of Glasgow
University of Gloucestershire
University of Hertfordshire
University of Hull
University of Leeds
University of Leicester
University of Lincoln
University of Liverpool
University of Manchester
University of Northampton
University of Nottingham
University of Oxford
University of Plymouth
University of Portsmouth
University of Reading
University of Sheffield
University of St Andrews
University of Sussex
University of Stirling
University College London (UCL)
University of East Anglia
Ulster University
University of Worcester

2.0 Online Survey Questions

The following sections are included, presented to participants chronologically:

- 2.1 Survey Introduction
- 2.2 Demographic Information
- 2.3 Media Statement (text written by the authors, with scientific information taken from Hansen (2007), Rahmstorf et al. (2007), Lenton (2011), Duarte et al. (2012), Spalding and Brown (2015), Westerling (2016)).
    - *2.3.1 Male Scientist One (M1) – Daniel Thompson*
    - *2.3.2 Male Scientist Two (M2) – Matthew Thompson*
    - *2.3.3 Female Scientist One (F1) – Rebecca Thompson*
    - *2.3.4 Female Scientist Two (F2) – Helen Thompson*
- 2.4 The Roles of a Scientist
- 2.5 Debrief

2.1 Survey Introduction

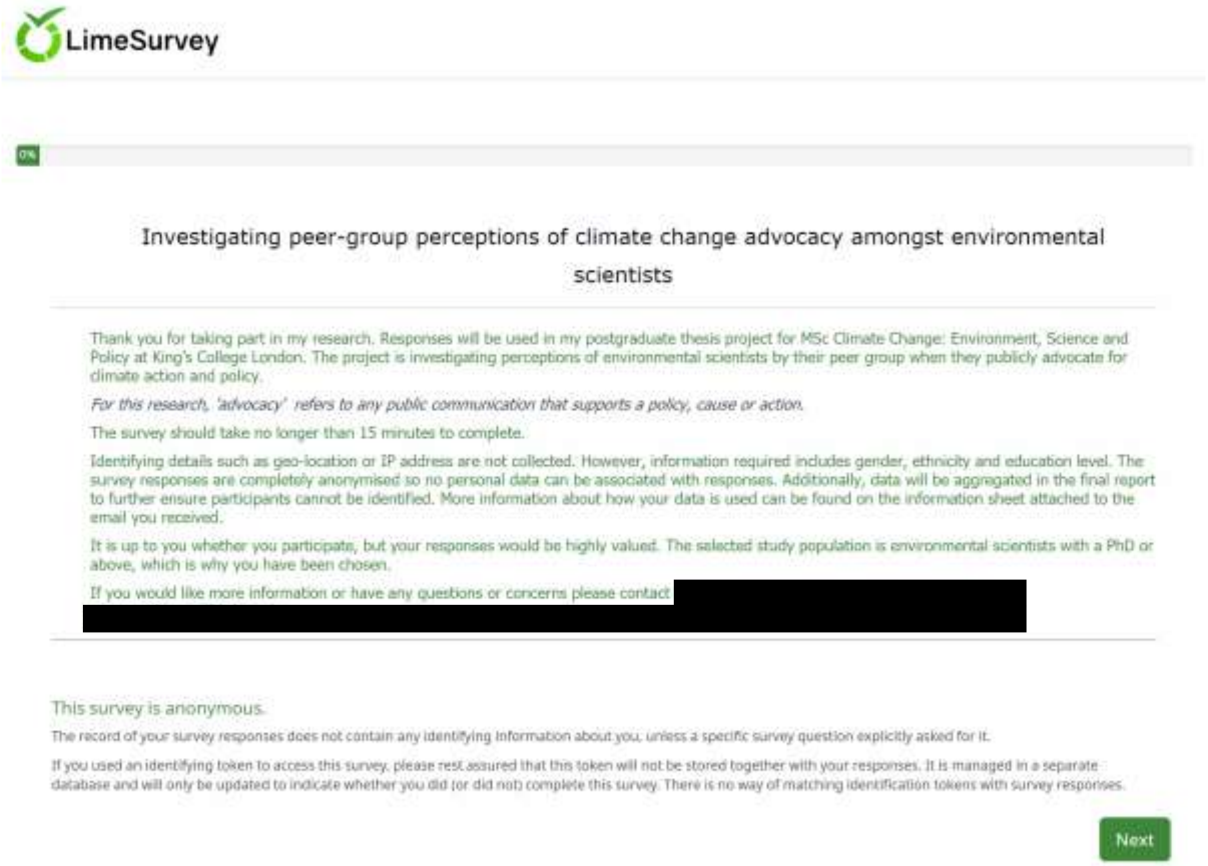

2.2 Demographic Information

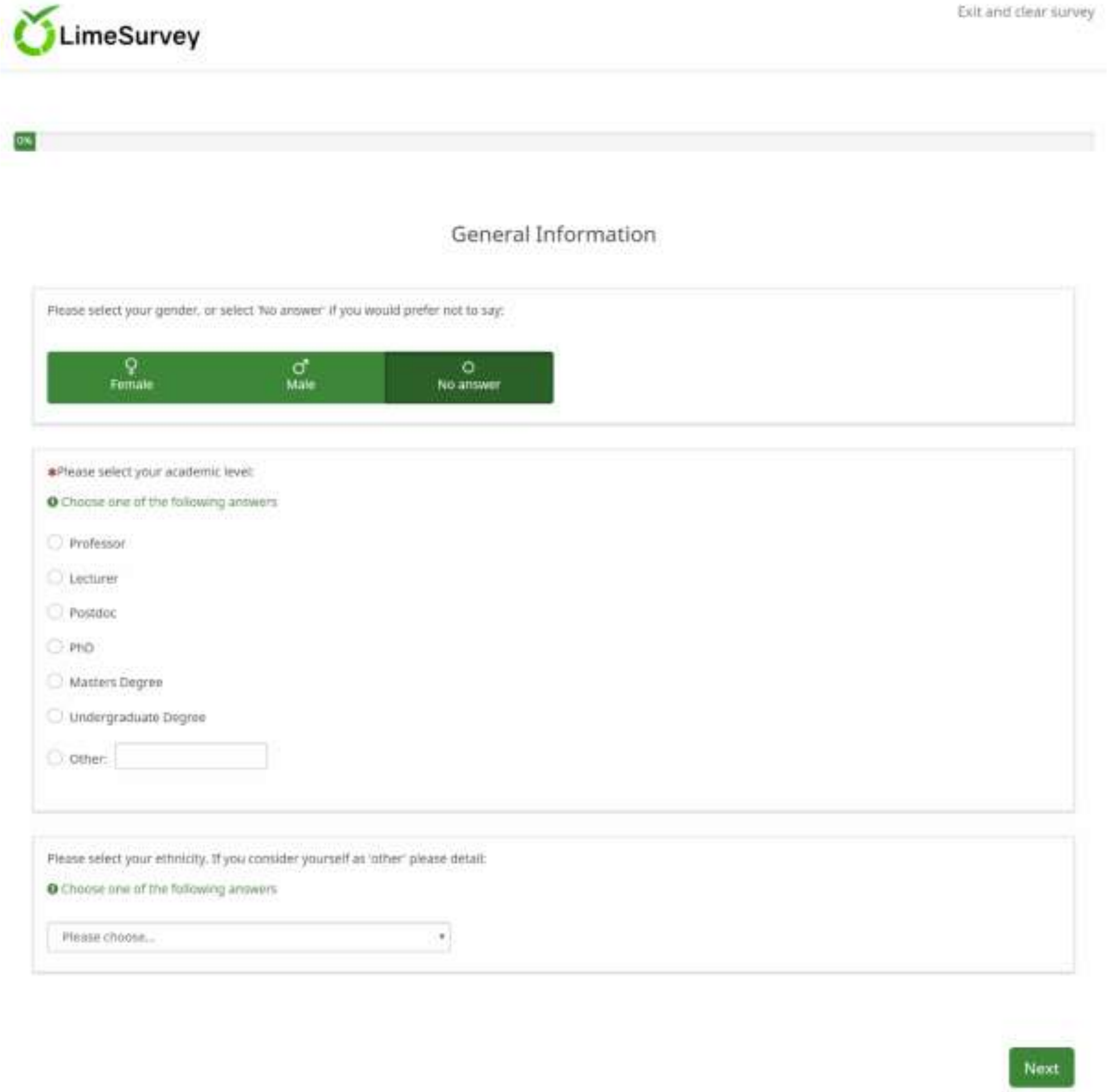

LimeSurvey

Exit and clear survey

0%

General Information

Please select your gender, or select 'No answer' if you would prefer not to say:

Female | Male | No answer

*Please select your academic level:

Choose one of the following answers

- Professor
- Lecturer
- Postdoc
- PhD
- Masters Degree
- Undergraduate Degree
- Other:

Please select your ethnicity. If you consider yourself as 'other' please detail:

Choose one of the following answers

Please choose...

Next

2.3 Media Statement

2.3.1 Male Scientist One (M1) – Daniel Thompson

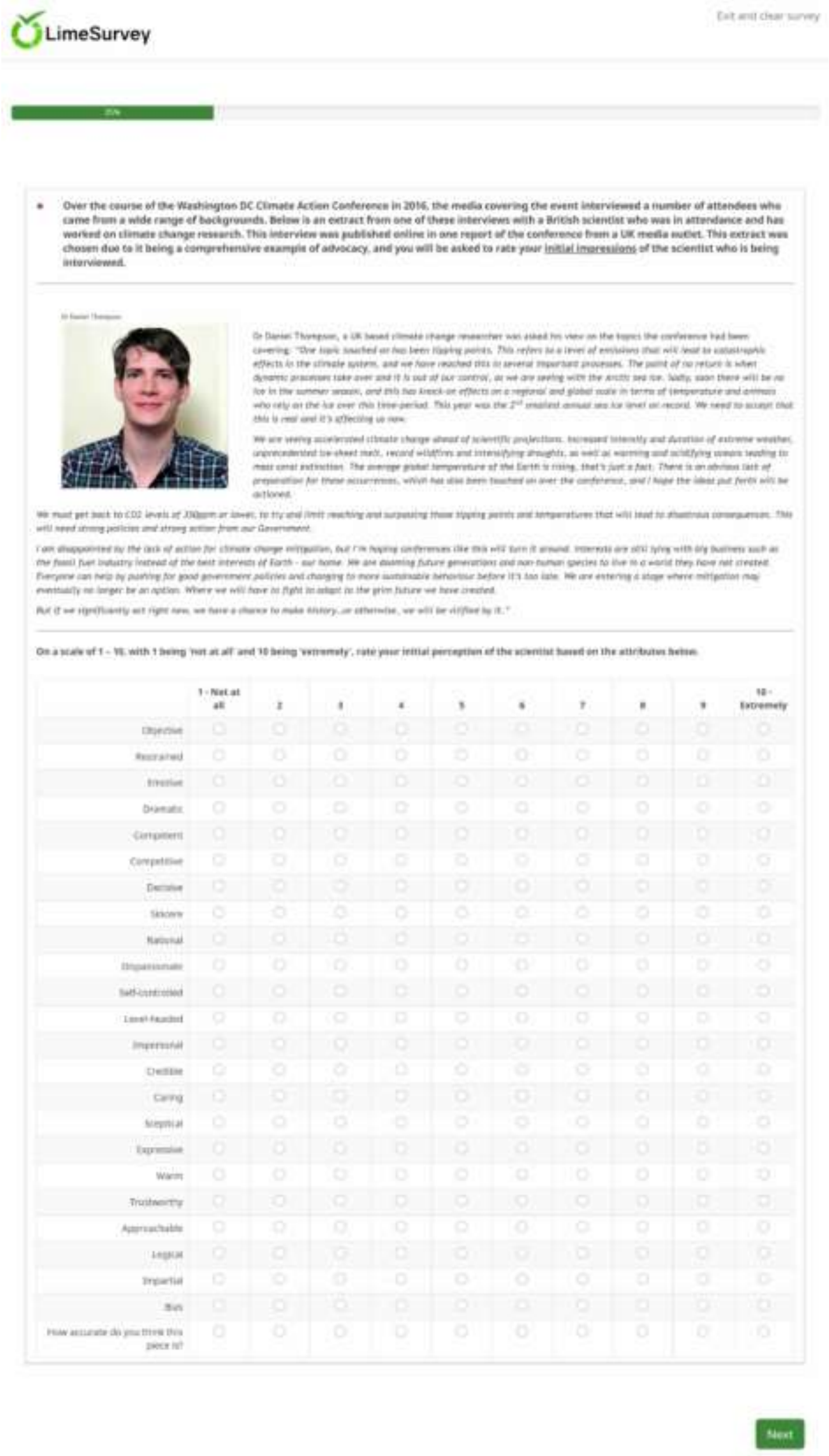

LimeSurvey

Exit and clear survey

25%

Over the course of the Washington DC Climate Action Conference in 2016, the media covering the event interviewed a number of attendees who came from a wide range of backgrounds. Below is an extract from one of these interviews with a British scientist who was in attendance and has worked on climate change research. This interview was published online in one report of the conference from a UK media outlet. This extract was chosen due to it being a comprehensive example of advocacy, and you will be asked to rate your initial impressions of the scientist who is being interviewed.

Dr Daniel Thompson

Dr Daniel Thompson, a UK based climate change researcher was asked his view on the topics the conference had been covering: *"One topic touched on has been tipping points. This refers to a level of emissions that will lead to catastrophic effects in the climate system, and we have reached this in several important processes. The point of no return is when dynamic processes take over and it is out of our control, as we are seeing with the Arctic sea ice. Sadly, soon there will be no ice in the summer season, and this has knock-on effects on a regional and global scale in terms of temperature and animals who rely on the ice over this time-period. This year was the 2nd smallest annual sea ice level on record. We need to accept that this is real and it's affecting us now.*

We are seeing accelerated climate change ahead of scientific projections. Increased intensity and duration of extreme weather, unprecedented ice-sheet melt, record wildfires and intensifying droughts, as well as warming and acidifying oceans leading to mass coral extinction. The average global temperature of the Earth is rising, that's just a fact. There is an obvious lack of preparation for these occurrences, which has also been touched on over the conference, and I hope the ideas put forth will be actioned.

We must get back to CO2 levels of 350ppm or lower, to try and limit reaching and surpassing those tipping points and temperatures that will lead to disastrous consequences. This will need strong policies and strong action from our Government.

I am disappointed by the lack of action for climate change mitigation, but I'm hoping conferences like this will turn it around. Interests are still lying with big business such as the fossil fuel industry instead of the best interests of Earth - our home. We are dooming future generations and non-human species to live in a world they have not created. Everyone can help by pushing for good government policies and changing to more sustainable behaviour before it's too late. We are entering a stage where mitigation may eventually no longer be an option. Where we will have to fight to adapt to the grim future we have created.

But if we significantly act right now, we have a chance to make history...or otherwise, we will be vilified by it."

On a scale of 1 – 10, with 1 being 'not at all' and 10 being 'extremely', rate your initial perception of the scientist based on the attributes below.

	1 - Not at all	2	3	4	5	6	7	8	9	10 - Extremely
Objective										
Restrained										
Emotive										
Dramatic										
Competent										
Competitive										
Decisive										
Sincere										
Rational										
Dispassionate										
Self-controlled										
Level-headed										
Impersonal										
Credible										
Caring										
Sceptical										
Expressive										
Warm										
Trustworthy										
Approachable										
Logical										
Impartial										
Bias										
How accurate do you think this piece is?										

Next

2.3.2 Male Scientist Two (M2) – Matthew Thompson

LimeSurvey

Over the course of the Washington DC Climate Action Conference in 2016, the media covering the event interviewed a number of attendees who came from a wide range of backgrounds. Below is an extract from one of these interviews with a British scientist who was in attendance and has worked on climate change research. This interview was published online in one report of the conference from a UK media outlet. This extract was chosen due to it being a comprehensive example of advocacy, and you will be asked to rate your <u>initial impressions</u> of the scientist who is being interviewed.

Dr Matthew Thompson

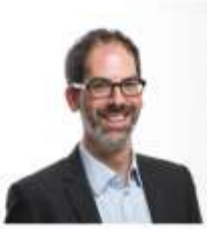

Matthew Thompson, a UK based climate change researcher was asked his view on the topics the conference had been covering: *"One topic touched on has been tipping points. This refers to a level of emissions that will lead to catastrophic effects in the climate system, and we have reached this in several important processes. The point of no return is when dynamic processes take over and it is out of our control, as we are seeing with the Arctic sea ice. Sadly, soon there will be no ice in the summer season, and this has knock-on effects on a regional and global scale in terms of temperature and animals who rely on the ice over this time-period. This year was the 2nd smallest annual sea ice level on record. We need to accept that this is real and it's affecting us now.*

We are seeing accelerated climate change ahead of scientific projections. Increased intensity and duration of extreme weather, unprecedented ice-sheet melt, record wildfires and intensifying droughts, as well as warming and acidifying oceans leading to mass coral extinction. The average global temperature of the Earth is rising, that's just a fact. There is an obvious lack of preparation for these occurrences, which has also been touched on over the conference, and I hope the ideas put forth will be actioned.

We must get back to CO2 levels of 350ppm or lower, to try and limit reaching and surpassing those tipping points and temperatures that will lead to disastrous consequences. This will need strong policies and strong action from our Government.

I am disappointed by the lack of action for climate change mitigation, but I'm hoping conferences like this will turn it around. Interests are still lying with big business such as the fossil fuel industry instead of the best interests of Earth - our home. We are dooming future generations and non-human species to live in a world they have not created. Everyone can help by pushing for good government policies and changing to more sustainable behaviour before it's too late. We are entering a stage where mitigation may eventually no longer be an option. Where we will have to fight to adapt to the grim future we have created.

But if we significantly act right now, we have a chance to make history...or otherwise, we will be vilified by it."

On a scale of 1 - 10, with 1 being 'not at all' and 10 being 'extremely', rate your initial perception of the scientist based on the attributes below.

	1 - Not at all	2	3	4	5	6	7	8	9	10 - Extremely
Objective										
Restrained										
Emotive										
Dramatic										
Competent										
Competitive										
Decisive										
Sincere										
Rational										
Dispassionate										
Self-controlled										
Level-headed										
Impersonal										
Credible										
Caring										
Sceptical										
Expressive										
Warm										
Trustworthy										
Approachable										
Logical										
Impartial										
Bias										
How accurate do you think this piece is?										

2.3.3 Female Scientist One (F1) – Rebecca Thompson

LimeSurvey

Over the course of the Washington DC Climate Action Conference in 2016, the media covering the event interviewed a number of attendees who came from a wide range of backgrounds. Below is an extract from one of these interviews with a British scientist who was in attendance and has worked on climate change research. This interview was published online in one report of the conference from a UK media outlet. This extract was chosen due to it being a comprehensive example of advocacy, and you will be asked to rate your <u>initial impressions</u> of the scientist who is being interviewed.

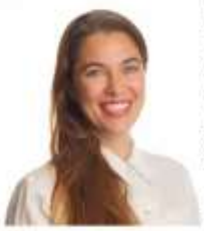

Dr Rebecca Thompson, a UK based climate change researcher was asked her view on the topics the conference had been covering: *"One topic touched on has been tipping points. This refers to a level of emissions that will lead to catastrophic effects in the climate system, and we have reached this in several important processes. The point of no return is when dynamic processes take over and it is out of our control, as we are seeing with the Arctic sea ice. Sadly, soon there will be no ice in the summer season, and this has knock-on effects on a regional and global scale in terms of temperature and animals who rely on the ice over this time-period. This year was the 2nd smallest annual sea ice level on record. We need to accept this is real and it's affecting us now.*

We are seeing accelerated climate change ahead of scientific projections. Increased intensity and duration of extreme weather, unprecedented ice-sheet melt, record wildfires and intensifying droughts, as well as warming and acidifying oceans leading to mass coral extinction. The average global temperature of the Earth is rising, that's just a fact. There is an obvious lack of preparation for these occurrences, which has also been touched on over the conference, and I hope the ideas put forth will be actioned.

We must get back to CO2 levels of 350ppm or lower, to try and limit reaching and surpassing those tipping points and temperatures that will lead to disastrous consequences. This will need strong policies and strong action from our Government.

I am disappointed by the lack of action for climate change mitigation, but I'm hoping conferences like this will turn it around. Interests are still lying with big business such as the fossil fuel industry instead of the best interests of Earth - our home. We are dooming future generations and non-human species to live in a world they have not created. Everyone can help by pushing for good government policies and changing to more sustainable behaviour before it's too late. We are entering a stage where mitigation may eventually no longer be an option. Where we will have to fight to adapt to the grim future we have created.

But if we significantly act right now, we have a chance to make history...or otherwise, we will be vilified by it."

On a scale of 1 - 10, with 1 being 'not at all' and 10 being 'extremely', rate your initial perception of the scientist based on the attributes below.

	1 - Not at all	2	3	4	5	6	7	8	9	10 - Extremely
Objective										
Restrained										
Emotive										
Dramatic										
Competent										
Competitive										
Decisive										
Sincere										
Rational										
Dispassionate										
Self-controlled										
Level-headed										
Impersonal										
Credible										
Caring										
Sceptical										
Expressive										
Warm										
Trustworthy										
Approachable										
Logical										
Impartial										
Bias										
How accurate do you think this piece is?										

2.3.4 Female Scientist Two (F2) – Helen Thompson

LimeSurvey

* **Over the course of the Washington DC Climate Action Conference in 2016, the media covering the event interviewed a number of attendees who came from a wide range of backgrounds. Below is an extract from one of these interviews with a British scientist who was in attendance and has worked on climate change research. This interview was published online in one report of the conference from a UK media outlet. This extract was chosen due to it being a comprehensive example of advocacy, and you will be asked to rate your <u>initial impressions</u> of the scientist who is being interviewed.**

Dr Helen Thompson

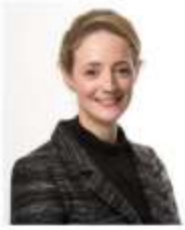

Dr Helen Thompson, a UK based climate change researcher was asked her view on the topics the conference had been covering: *"One topic touched on has been tipping points. This refers to a level of emissions that will lead to catastrophic effects in the climate system, and we have reached this in several important processes. The point of no return is when dynamic processes take over and it is out of our control, as we are seeing with the Arctic sea ice. Sadly, soon there will be no ice in the summer season, and this has knock-on effects on a regional and global scale in terms of temperature and animals who rely on the ice over this time-period. This year was the 2nd smallest annual sea ice level on record. We need to accept that this is real and it's affecting us now.*

We are seeing accelerated climate change ahead of scientific projections. Increased intensity and duration of extreme weather, unprecedented ice-sheet melt, record wildfires and intensifying droughts, as well as warming and acidifying oceans leading to mass coral extinction. The average global temperature of the Earth is rising, that's just a fact. There is an obvious lack of preparation for these occurrences, which has also been touched on over the conference, and I hope the ideas put forth will be actioned.

We must get back to CO2 levels of 350ppm or lower, to try and limit reaching and surpassing those tipping points and temperatures that will lead to disastrous consequences. This will need strong policies and strong action from our Government.

I am disappointed by the lack of action for climate change mitigation, but I'm hoping conferences like this will turn it around. Interests are still lying with big business such as the fossil fuel industry instead of the best interests of Earth - our home. We are dooming future generations and non-human species to live in a world they have not created. Everyone can help by pushing for good government policies and changing to more sustainable behaviour before it's too late. We are entering a stage where mitigation may eventually no longer be an option. Where we will have to fight to adapt to the grim future we have created.

But if we significantly act right now, we have a chance to make history...or otherwise, we will be vilified by it."

On a scale of 1 – 10, with 1 being 'not at all' and 10 being 'extremely', rate your initial perception of the scientist based on the attributes below.

	1 - Not at all	2	3	4	5	6	7	8	9	10 - Extremely
Objective	○	○	○	○	○	○	○	○	○	○
Restrained	○	○	○	○	○	○	○	○	○	○
Emotive	○	○	○	○	○	○	○	○	○	○
Dramatic	○	○	○	○	○	○	○	○	○	○
Competent	○	○	○	○	○	○	○	○	○	○
Competitive	○	○	○	○	○	○	○	○	○	○
Decisive	○	○	○	○	○	○	○	○	○	○
Sincere	○	○	○	○	○	○	○	○	○	○
Rational	○	○	○	○	○	○	○	○	○	○
Dispassionate	○	○	○	○	○	○	○	○	○	○
Self-controlled	○	○	○	○	○	○	○	○	○	○
Level-headed	○	○	○	○	○	○	○	○	○	○
Impersonal	○	○	○	○	○	○	○	○	○	○
Credible	○	○	○	○	○	○	○	○	○	○
Caring	○	○	○	○	○	○	○	○	○	○
Sceptical	○	○	○	○	○	○	○	○	○	○
Expressive	○	○	○	○	○	○	○	○	○	○
Warm	○	○	○	○	○	○	○	○	○	○
Trustworthy	○	○	○	○	○	○	○	○	○	○
Approachable	○	○	○	○	○	○	○	○	○	○
Logical	○	○	○	○	○	○	○	○	○	○
Impartial	○	○	○	○	○	○	○	○	○	○
Bias	○	○	○	○	○	○	○	○	○	○
How accurate do you think this piece is?	○	○	○	○	○	○	○	○	○	○

2.4 The Roles of a Scientist

LimeSurvey

Exit and clear survey

50%

* Below are 5 idealised roles scientists can play in science, advocating and communication adapted from Pielke (2007) and Rapley (2014).

Please select the role you think scientists should ideally have in relation to climate change discourse, and the role you would be comfortable with yourself (for this exercise, please assume your work has relevance to the climate change discourse).

	Scientists should ideally have this role	I would be comfortable with this role
**Pure Scientist** - focuses solely on generating 'facts' and delivering them to the 'pool of human knowledge', with no consideration for their use or utility, and no direct connection with decision-makers, who are left to find out for themselves what they need to know.	○	○
**Science Communicator** – engages with society to make aware and discuss the results and implications of the research that it has funded, including the task of raising the alert if the implications of a piece of research point to a significant societal threat or opportunity. This includes explicitly engaging with public discourse and collaboration with social scientists to ensure evidence-based approach to communication.	○	○
**Science Arbiter** - seeks to stay removed from explicit considerations of policy and politics but answers factual questions posed by a decision-maker. A key characteristic is to avoid questions that cannot be resolved by scientific enquiry.	○	○
**Issue Advocate** - engages with a decision-maker and public seeking to reduce the scope of choice available by promoting a particular course of action that they justify using their expert knowledge and understanding.	○	○
**Honest Broker of Policy Alternatives** - engages in decision-making, contributing knowledge and understanding collaboratively with other participants (such as other schools of academia and the public) to expand and clarify the scope of choice available, and to converge collectively on an agreed way forward.	○	○

If you have any additional comments, please add them here.

Next

2.5 Debrief

LimeSurvey

Thank you for your participation in my study! Please read the below before closing your browser.

Purpose of the Study

Earlier in the study, I informed you that the purpose of the research was investigating peer-group perceptions of climate change advocacy amongst environmental scientists. In actuality, my study is about how potential implicit gender bias might influence perceived attributes of environmental scientists acting as advocates by their peers. You have been shown an advocacy piece that was randomly assigned either a female or male image and name. The gender aspect was not mentioned at the beginning of the study, and the articles and images used were fabricated. The results will be used to investigate bias that could be present based on the influence of gender and scientific norms in society and academia.

Unfortunately, in order to properly test my hypothesis, I could not provide you with all of these details prior to your participation. This ensures that your reactions in this study were spontaneous and not influenced by prior knowledge about the purpose of the study. The initial information about the study was not entirely accurate, and a fabricated advocacy piece was used in conjunction with a randomized selection of a fictitious male or female scientist to investigate the real purpose of the study; the influence of gender on peer perception of scientists acting as advocates, specifically relating to climate change. If I had told you the actual purposes of my study, it would be unlikely any unconscious and implicit bias could be reliably investigated in the responses. Implicit bias comes from repeated contact with ingrained cultural stereotypes and is an automatic bias that affects thoughts and actions in unconscious ways. This needed to be measured without self-mediation by participants. I regret the deception, but I hope you understand the reason for it.

Your results are anonymous, and data will be aggregated in the final report to further ensure responses cannot be matched with participants.

Why is this important to study?

It is important for scientists to be involved in the talks around climate change and communicate their work and ideas to the public and decision makers to engage, educate and foster democratic discussion in the context of current knowledge and uncertainties. There is an ongoing reticence which can be ascribed to scientific norms, fear of backlash and loss of credibility. Sticking to these norms allow scientists to avoid losing standing within their peer group and the public. Many core values of science are often seen as 'masculine' and it is often viewed as inappropriate to have emotive attributes that appear at odds with the objectivity of science. This is worrying from a gender perspective for female scientists in the public eye, as female attribute stereotypes tend not to be associated with science, and combined with the emotive realm of media, may lead to tougher judgments.
If peers view female scientists as less credible or more negatively if they enter the public sphere - explicitly or implicitly - this has the potential to be detrimental to the retention of women in science, reinforce implicit bias, lead to professional issues and peer rejection, and elicit reticence in a portion of the scientific community who will communicate the importance of climate change action to the public. Additionally, it will ultimately cut the diversity of perspectives that are needed to create a more complex picture of the world in scientific exploration and innovation.

Confidentiality and how your data will be used

Please note that although the purpose of this study is different from the originally stated purpose, everything else on the information sheet that was attached to the original email is correct. This includes the ways in which your data is kept confidential. This survey was used to collect research data for my dissertation, including general information about gender, education level and ethnicity, and responses about advocacy. Your input will be used to investigate whether there is an implicit gender bias based on attributes against those who advocate publicly about climate change. No names, cookies or IP information was collected as part of this research. Every response has been anonymised by the survey software 'LimeSurvey' and unidentifiable to the researcher. The responses will be stored anonymously on the survey software and the KCL OneDrive of the researcher for the duration of the research. Both are password protected. Study data will be stored with King's College London for one year after the completion of the research. Any email addresses used to contact participants are kept separately from responses in a password protected file and will be deleted within 3 months of the projects submission date. This data will be solely used by the researcher and not shared.

Now the study is complete, it is not possible to identify and delete your responses. This is because the results are anonymised; so single surveys will not be possible to identify. All responses have been anonymized and will be aggregated and presented as a group in the final report to ensure responses cannot be matched with participants.

I would appreciate if you would not disclose the research procedures and/or hypotheses to anyone who might participate in this study in the future as this could affect the results of the study.

Useful Contact Information

If you have any questions or concerns regarding this study, its purpose or procedures, please feel free to contact:

[redacted]

*****Please copy this text if you would like it for future reference or contact the above. Once again, thank you for your participation in this study! *****

3.0 Breakdown of Results: Participant Demographics

Table S1: Participant demographic information, broken down by the scientist assigned to each participant.

Demographic	M1	M2	F1	F2	Total
**Total Participants**	21	16	27	27	91
**Participant Gender**					
Female	7	3	7	6	23
Male	11	10	17	19	57
Prefer not to say	3	3	3	2	11
**Academic Level**					
PhD	1	-	-	2	3
Post Doc	2	6	7	6	21
Lecturer	13	8	10	11	42
Professor	5	2	10	8	25
**Ethnicity**					
White – British	13	10	21	17	61
White – Irish	1	-	-	2	3
Asian / Asian British – Indian	-	-	-	1	1
Asian / Asian British – Pakistani	1	-	-	-	1
Other – European / European British	2	3	-	4	10
Other – White (not stated)	2	3	5	2	11
Other – White American	-	-	-	1	1
Other – Jewish	-	-	1	-	1
Other – Not stated	2	-	-	-	2

4.0 Breakdown of Results: Gender Perceptions

Table S2: P-values of permutation tests to assess differences in rankings between the fictitious scientists: MC (n=37), FC (n=54), M1 (n=21), M2 (n=16), F1 (n=27) and F2 (n=27). P-values are highlighted where significant to at least p ≤ 0.1.

Attributes	P-Values for Comparisons						
	MC vs FC	F1 vs F2	F1 vs M1	F1 vs M2	F2 vs M1	F2 vs M2	M1 vs M2
**Objective**	1	0.21	1	0.32	0.77	0.99	0.75
**Restrained**	1	1	0.26	1	0.4	1	0.74
**Emotive**	0.11	0.28	0.78	0.4	0.13	0.14	0.53
**Dramatic**	1	0.13	0.57	0.58	0.57	0.14	0.99
**Competent**	1	0.15	1	1	0.27	0.35	0.1
**Competitive**	0.17	0.61	0.77	0.27	0.43	0.2	0.88
**Decisive**	1	1	0.57	1	1	0.53	0.74
**Sincere**	0.82	0.79	1	0.51	0.78	1	0.49
**Rational**	1	1	1	0.16	1	0.39	0.15
**Dispassionate**	1	0.78	0.78	1	0.08*	0.56	0.74
**Self-controlled**	0.66	0.78	1	0.55	0.42	1	0.44
**Level-headed**	1	0.61	1	0.55	0.45	0.77	0.34
**Impersonal**	0.08	0.57	1	1	0.36	0.37	0.33
**Credible**	1	0.21	1	1	0.27	0.75	1
**Caring**	1	1	1	0.26	1	0.76	0.49
**Sceptical**	1	0.27	1	0.21	1	0.21	0.98
**Expressive**	0.4	1	1	0.53	1	0.34	0.51
**Warm**	0.69	0.39	1	1	0.32	0.28	1
**Trustworthy**	0.67	1	1	0.76	1	0.53	0.74
**Approachable**	0.82	0.79	0.78	0.7	1	0.25	0.16
**Logical**	1	0.15	1	0.06*	0.39	0.21	0.08
**Impartial**	1	0.59	1	0.43	0.76	1	0.49
**Biased**	1	0.34	0.39	0.3	0.57	0.98	0.54
**Accurate**	1	1	1	0.58	1	0.54	0.78

* p ≤ 0.1

Table S3. As Table S2 but for the combined attribute scales designed to represent stereotypical associations with ‘Science’, ‘Media’, ‘Male’ and ‘Female’.

**Comparison (X vs Y)**	**Stereotypical Attribute Scales**			
	**‘Science’**	**‘Media’**	**‘Male’**	**‘Female’**
**MC vs FC**	p = 1	p = 0.47	p = 1	p = 0.27
**F1 vs F2**	p = 1	p = 0.34	p = 0.68	p = 1
**F1 vs M1**	p = 1	p = 1	p = 1	p = 1
**F1 vs M2**	p = 0.004††[1]	p = 1	p = 0.35	p = 0.63
**F2 vs M1**	p = 1	p = 0.25	p = 0.86	p = 0.28
**F2 vs M2**	p = 0.78	p = 0.37	p = 1	p = 0.12
**M1 vs M2**	p = 0.006††[2]	p = 1	p = 0.76	p = 1

† p ≤ 0.05
†† p ≤ 0.01

1. Y>X = 52% (46-56%), Y<X = 35% (30-41%), Y=X = 13%
2. Y>X = 53% (7-58%), Y<X = 33% (28-39%), Y=X = 14%

References

Duarte CM, Lenton TM, Wadhams P, et al. (2012) Abrupt climate change in the Arctic. *Nature Climate Change* 2(2). 2. Nature Publishing Group: 60–62. DOI: 10.1038/nclimate1386.

Hansen JE (2007) Scientific reticence and sea level rise. *Environmental Research Letters* 2(2). IOP Publishing: 024002. DOI: 10.1088/1748-9326/2/2/024002.

Lenton TM (2011) Early warning of climate tipping points. *Nature Climate Change* 1(4). 4. Nature Publishing Group: 201–209. DOI: 10.1038/nclimate1143.

Rahmstorf S, Cazenave A, Church JA, et al. (2007) Recent Climate Observations Compared to Projections. *Science* 316(5825): 709–709. DOI: 10.1126/science.1136843.

Spalding MD and Brown BE (2015) Warm-water coral reefs and climate change. *Science* 350(6262): 769–771. DOI: 10.1126/science.aad0349.

Westerling AL (2016) Increasing western US forest wildfire activity: sensitivity to changes in the timing of spring. *Philosophical Transactions of the Royal Society B: Biological Sciences* 371(1696): 20150178. DOI: 10.1098/rstb.2015.0178.
